# Effects of Epigallocatechin-3-gallate (EGCG) on the bond strength of fiber posts to Sodium hypochlorite (NaOCl) treated intraradicular dentin

**DOI:** 10.1038/s41598-017-04107-8

**Published:** 2017-06-26

**Authors:** Hao-han Yu, Ling Zhang, Shuai Xu, Fang Li, Fan Yu, Zheng-ya Liu, Li Huang, Ji-hua Chen

**Affiliations:** 1 State Key Laboratory of Military Stomatology & National Clinical Research Centre for Oral Disease & Shaanxi key Laboratory of Stomatology, Department of Prosthodontics, School of Stomatology, Fourth Military Medical University, Xi’an, 710032 China; 2 Department of prosthodontics, Xinqiao hospital, Third Military Medical University, Chongqing, 400037 China; 3 State Key Laboratory of Military Stomatology & National Clinical Research Centre for Oral Disease & Shaanxi key Laboratory of Oral Disease, Department of General Dentistry and Emergency, School of Stomatology, Fourth Military Medical University, Xi’an, Shaanxi 710032 China

## Abstract

This study was to evaluate the effect of Epigallocatechin-3-gallate (EGCG) on the bond strength of two adhesive systems to the Sodium hypochlorite (NaOCl) treated intraradicular dentin. The roots were accepted regular root canal treatments and post space preparations, and further divided into eight groups according to the four post space pretreatments and two dentin adhesives [Single Bond 2 (SB2) and Clearfil SE Bond (CSB)] used. The push-out strength before and after thermocycling in different root region (coronal and apical), DC of the adhesive and morphologic patterns of treated post space were evaluated. NaOCl + EGCG groups showed the highest push-out strength regardless of the adhesive type, root region and time (*P* < *0.05*). NaOCl pretreatment significantly decreased the push-out strengths and DC of CSB (*P* < *0.05*). EGCG could improve the bonding properties of both SB2 and CSB to NaOCl treated intraradicular dentin. The effect of NaOCl on bonding of a fiber post depended on the type of the adhesive.

## Introduction

Recently years, fiber post has been popularly used to restore endodontically treated teeth with extensive loss of coronal tooth structure^1^. Fiber posts are friendly welcome by both patients and clinical dentists for their aesthetic properties, favorable biomechanical properties and the convenient clinical procedures^2–4^. However, both *in vivo* and *in vitro* studies have shown that the loss of retention of fiber posts from root canals is the dominant failure to limit the long-term performance of endodontically teeth using post-core restorations^3–6^.

Overall, the retention of fiber posts is dependent on the firm and lasting adhesion between the resin cement and the dentine, as well as on the adhesion between the resin cement and posts^7^. Well bonding is based on the formation of so-called hybrid layer (HL) with resin monomer infiltration into the demineralized dentin, which requires a clean dentinal surface after mechanical post space preparation with the removal of root canal filling material^8^. However, a routine post space preparation always produces a new smear layer rich in sealer and gutta-percha remnants that plasticized by the friction heat of the drill^9^. The thick smear layer on the post space would impair the effective bonding of adhesive resin to intraradicular dentin^10^. Apart from effective immediate bonding, acquiring the long-term bond stability of current resin bonding materials and dentin gained immense attention^11, 12^. The endogenous matrix metalloproteinases (MMPs) and cysteine cathepsins have been reported to play an important role in the degradation of dentin bond strength^13, 14^.

Etching, chemical irrigation, ultrasonic treatment and other methods have been evaluated to remove the smear layer on post spaces but showed controversial results^15–18^. Among the irrigation protocols, irrigation with sodium hypochlorite (NaOCl) solution is one of the mostly used methods due to its properties of dissolving organic tissues, saponifying fats and neutralizing toxic products and antibacterial^19^. NaOCl treatment on dentin surfaces has been reported to be effective on deproteinizing the dentin surface and improving the adhesive wettability^20^. Therefore, some researchers have hypothesized that the collagen removal with NaOCl might contribute to long-term adhesion stability of resin-dentin^21, 22^. However, the use of NaOCl on post space pretreatment was also problematic considering its negative effect on the polymerization of the adhesive resin^23^. However, no direct evidence was given in former studies. The adhesive properties of adhesives are determined by the degree of monomer conversion. The non-reacted double-bonds provide the parameter to assess the materials degree of conversion (DC)^24^. Micro-Raman spectroscopy has been proved an effective method to evaluate the DC of adhesives^24^.

Biocompatible reducing agents, such as ascorbic acid and sodium ascorbate, have seemed to be promising on reversing the negative effects of NaOCl to improve the bonding performance of fiber post in root canals and can be explained by the antioxidant ability of ascorbic acid^25–28^. Epigallocatechin-3-gallate (EGCG) is the major polyphenolic constituent found in green tea and have been reported to have several biochemical functions^25, 26, 29^. Former studies proved that EGCG could protect the organic matrix of dentin from demineralization attack and improve the durability of resin-dentin bonds^30–35^. Besides, EGCG is a strong anti-oxidant^29^ which make it possible to work as a reducing agent, like sodium ascorbate, to neutralize residual NaOCl via a redox reaction. However, there was no report to use about EGCG as a post space pretreatment when bonding a fiber post.

The purpose of the present study was to test the effect of Epigallocatechin-3-gallate (EGCG) on the bond strength of two adhesive systems to the Sodium hypochlorite (NaOCl) treated intraradicular dentin. A self-etching dentin adhesive and an etch-and-rinse were used in the present study. The null hypotheses to be tested were as the following: (1) EGCG has no effect on bond strength of fiber-post to NaOCl treated intraradicular dentin; (2) the two bonding systems show no difference in their bonding performance in intraradicular dentin.

## Results

### Push-out strength

Four-way ANOVA showed that the type of adhesives, post space pretreatments, root regions and time all showed a significant effect on the push-out strength (*P* < 0.001). Interaction between type of adhesives and post space pretreatments (*P* < 0.001), between type of adhesives and root regions (*P* = 0.049), between root regions and post space pretreatments (P = 0.005) and between post space pretreatments and time (P = 0.014) were both significant. The push-out strength of all tested groups was summarized in Table 1. The bond strength of NaOCl + EGCG (NEG) group was significantly higher than NaOCl (NA) group, regardless of type of adhesives, root region and time. After thermocycling, the bond strength of all groups decreased, except for NEG group.

**Table 1:** Push-out strength values (n = 24; MPa; mean ± standard deviation).

Post-space pretreatment	CSB				SB2			
	Coronal		Apical		Coronal		Apical	
	IM	AT	IM	AT	IM	AT	IM	AT
BC	8.45 ± 2.00^1,2,a,A,^*	5.13 ± 1.86^1,a,A,^**	6.42 ± 2.03^1,2,b,A,^*	3.23 ± 1.45^1,a,^**	12.95 ± 3.03^1,a,B,^*	8.31 ± 2.13^1,a,B,^**	7.65 ± 1.93^1,b,A,^*	5.01 ± 1.45^1,b,^**
NA	6.08 ± 1.56^1,a,A,^*	3.13 ± 1.24^1,a,A,^**	4.01 ± 1.39^1,b,A,^*	1.73 ± 0.92^1,a,A,^**	13.62 ± 1.88^1,a,B,c^	9.02 ± 1.56^1,a,A,^**	8.47 ± 0.74^1,b,B,^*	5.93 ± 1.21^1,2,b,B,^**
NEG	10.80 ± 1.30^2,a,A,^*	8.92 ± 1.56^2,a,A,^*	6.69 ± 1.15^2,b,A,^*	5.88 ± 1.47^2,b,A,^*	17.65 ± 3.38^2,a,B,^*	16.01 ± 2.23^3,a,B,^*	10.85 ± 1.78^2,b,B,^*	9.13 ± 1.52^3,b,B,^*
NE	7.12 ± 1.09^1,a,A,^*	4.96 ± 1.24^1,a,A,^**	4.98 ± 1.52^1,2,b,A,^*	2.88 ± 1.37^1,b,A,^**	14.31 ± 1.47^1,2,a,B,^*	12.13 ± 1.64^2,a,B,^**	9.02 ± 1.01^1,2,b,B,^*	7.89 ± 1.32^2,3,b,B,^*

*For each vertical column, values with identical numbers indicate no significant difference (P > 0.05). For each horizontal row, values with identical lowercase letters indicate no significant difference between root regions within the same adhesive system and the same time (P > 0.05), values with identical uppercase letters indicate no significant difference between adhesive systems within the same root region and the same time (P > 0.05), and values with identical number of asterisks indicate no significant difference between time within the same adhesive system and the same root region. IM, Immediate; AT, After thermocycling.

The distributions of failure mode were summarized in Table 2. The chi-square test revealed that there were significant differences in the failure mode within all tested groups (*P* < *0.05*). Regardless of adhesives and time, NA group showed the significantly highest percentage of adhesive failure between dentin and adhesives, followed by Blank Control (BC) group, NaOCl + Ethanol (NE) group and NEG group in turn (*P* < 0.05).

**Table 2:** Distribution of failure mode (n = 24).

Adhesives	Post Space Treatment	Time	Failure Mode				
			i	ii	iii	iv	v
CSB	BC	IM	1	5	0	0	18
		AT	2	8	0	0	14
	NA	IM	1	8	1	0	14
		AT	1	10	0	1	12
	NEG	IM	0	2	0	0	22
		AT	1	2	0	0	21
	NE	IM	2	4	1	2	15
		AT	1	5	0	3	15
SB2	BC	IM	2	5	1	1	15
		AT	1	7	0	0	16
	NA	IM	0	7	2	1	14
		AT	1	10	1	1	11
	NEG	IM	1	2	0	1	20
		AT	0	3	0	0	21
	NE	IM	1	3	0	2	18
		AT	1	7	0	0	16

IM, immediate; AT, after thermocycling. Failure mode: (i) adhesive failure between post and cement; (ii) adhesive failure between dentin and cement; (iii) cohesive failure within cement; (iv) cohesive failure within dentin; and (v) mixed failure.

### Micro-Raman analysis for DC measurement

**Figure 1:**
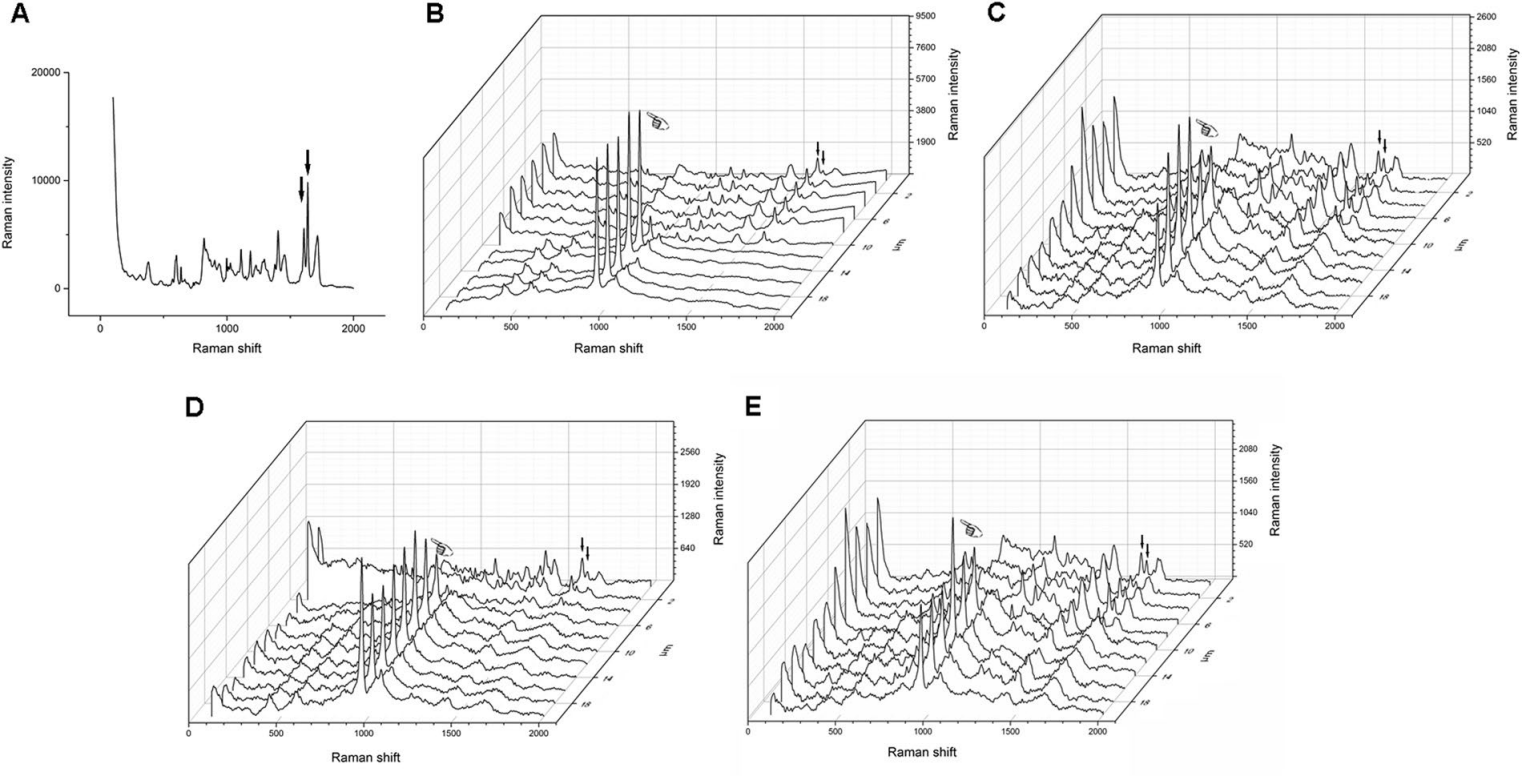
Micro-Raman spectrum acquired with CSB. (**A**) The representive spectrum of uncured CSB. The representive spectrum acquired across the adhesive–dentine interface produced by cured CSB of three groups: B-BC Group; C-NA Group; D, NEG Group; E, NE Group. In all figures, the first spectrum was collected in mineralized dentine: the finger indicated the PO_4_^3−^ group. The simultaneous decrease in the phosphate peak and increases in the adhesive peaks at 1608 cm^−1^ and at 1640 cm^−1^ (arrows) suggested the beginning of the hybrid layer.

**Figure 2:**
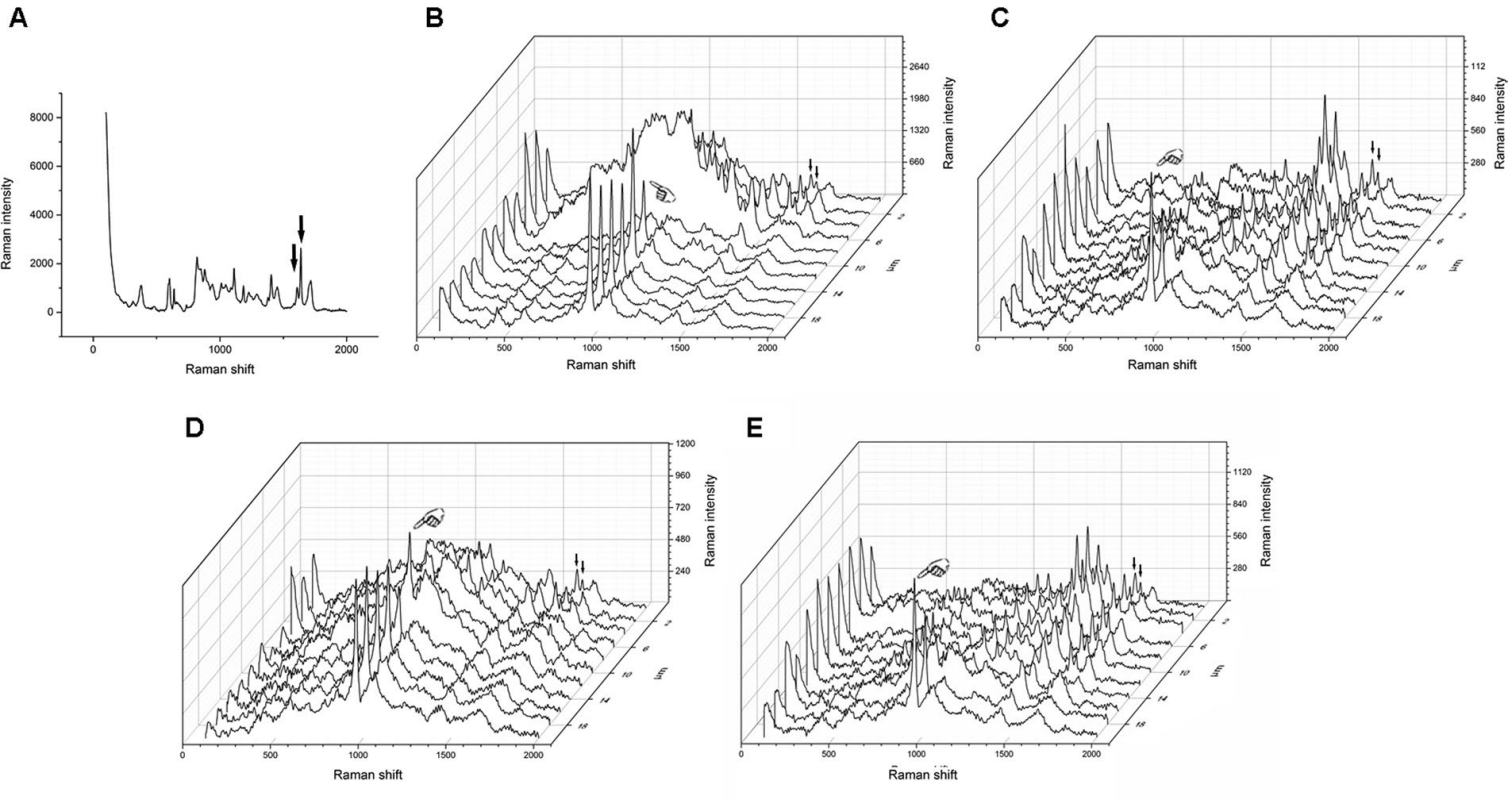
Micro-Raman spectrum acquired with SB2. (**A**) The representive spectrum of uncured SB2. The representive spectrum acquired with across the adhesive–dentine interface produced by cured SB2 of three groups: B-BC Group; C-NA Group; D, NEG Group; E, NE Group. In all figures, the first spectrum was collected in mineralized dentine: the finger indicated the PO_4_^3−^ group. The simultaneous decrease in the phosphate peak and increases in the adhesive peaks at 1608 cm^−1^ and at 1640 cm^−1^ (arrows) suggested the beginning of the hybrid layer.

**Figure 3:**
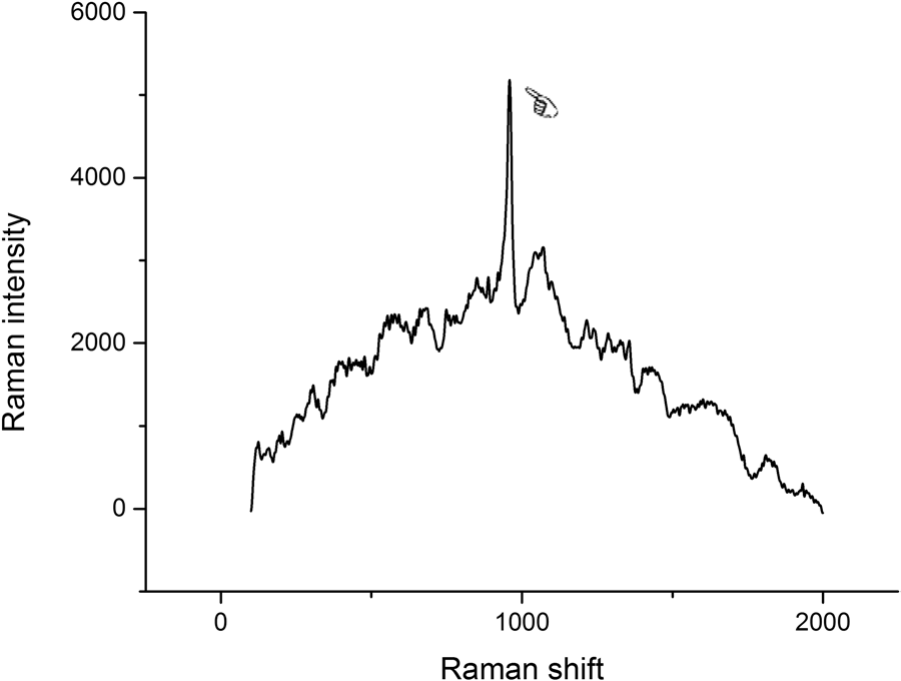
Representative micro-Raman spectrum of mineralized dentine. At 960 cm^−1^, the peak is associated with the mineral PO_4_^3−^ group is clearly visible (finger).

Representative Raman spectrums of the uncured and cured Raman spectrums of Clearfil SE Bond (CSB) and Single Bond 2 (SB2) were presented in Figs 1 and 2 respectively. The Raman intensity at 1640 cm^−1^ and 1608 cm^−1^ (indicated by the arrows in Figs 1 and 2) were employed to determine the DC of the adhesive. The representative Raman spectrum of dentin was presented in Fig. 3 and the Raman intensity at 960 cm^−1^ is associated with the mineral PO_4_^3−^ group.

The mean values of DC of two adhesives after different post space pretreatments are shown in Table 3. For CSB, the DC of NA group and NEG group was significantly lower than those of BC group and NEG group, while the DC in the former two groups and later two groups showed no significantly differences *(P* < *0.05)*. Different from CSB, the post space pretreatments showed no significant effect on the DC of SB2 *(P* > *0.05*).

**Table 3:** Conversion degree values (n = 5; %; mean ± standard deviation).

Post-space pretreatment	CSB	SB2
BC	72.63 ± 1.47^a^	72.06 ± 1.62^A^
NA	58.51 ± 4.36^b^	70.73 ± 1.68^A^
NEG	70.89 ± 3.94^a^	72.03 ± 0.65^A^
NE	57.37 ± 3.82^b^	71.03 ± 1.33^A^

For each vertical column, means with the same superscript letter are not significantly by Tukey test (*P* > 0.05).

### SEM observation

**Figure 4:**
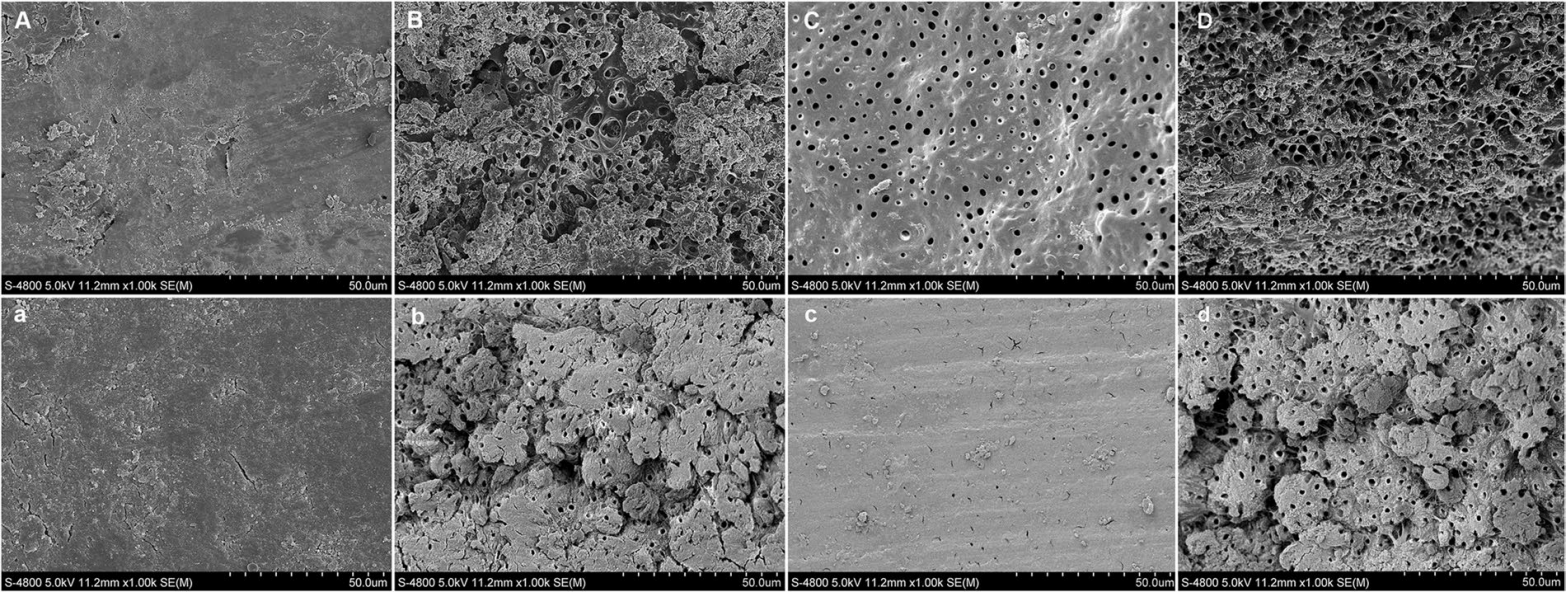
SEM photomicrographs of post space surfaces after different treatments (×1,000). In BC group (**A** and **a**), the post space surfaces were covered by thick smear layers. In NA group (**B** and **b**) and NE group (**D** and **d**), large amount of smear layer could still be observed on the dentin surfaces with small part of dentinal tubules being opened. In NEG group (**C** and **c**), the surfaces of post spaces were cleaner with only partial of smear layer remained. Higher degree of smear layers could be detected in apical region than in coronal region (**A–D**).

**Figure 5:**
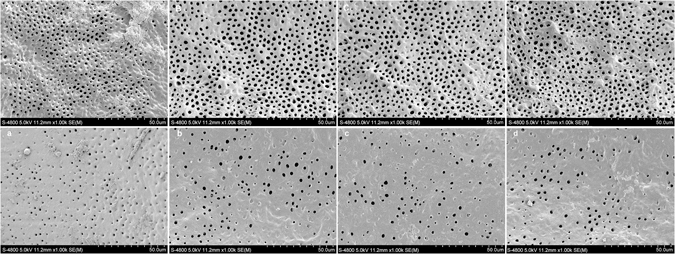
SEM photomicrographs of post space surfaces after treatments with different irrigants and phosphoric acid etching (×1,000). Only small part of smear layer remained on the post spaces of all groups. Compared with PA + BC groups (**A** and **a**), PA + NA groups (**B** and **b**) showed higher level of demineralization on dentin surfaces with wider opening of the dentinal tubule orifices. Less smear layer was covered in coronal regions (**A** and **B**) with wider opening of dentinal tubules than in apical region (**a** and **b**). PA + NEG groups (**C** and **c**) and PA + NE (**D** and **d**) groups showed similar to PA + NA groups.

The ultrastructures of post space surfaces after different treatments were presented in Figs 4 and 5. For CSB, the post space surfaces were covered by a thick smear layer regardless of the root region in BC group (Fig. 4A and a). The structure of dentinal tubules was hardly to be detected. In NA group, large amount of smear layer could still be observed on the dentin surfaces with partial dentinal tubules being detected along with opening dentinal tubule orifices (Fig. 4B). Compared with coronal region, more debris was left on the surfaces of apical region (Fig. 4b), and smaller diameter of opened dentinal tubule orifices in apical regions was observed. In NEG group, the surfaces of post spaces were cleaner than NA group, with only partial smear layer remained in both coronal and apical region (Fig. 4C and c). The degree of demineralization and deproteination of NEG group was less than that of NA group: more peritubular dentin was remained in NEG group and smaller diameter of tubule orifices than those in NA group (Fig. 4). The morphological presentations of NE group (Fig. 4D and d) were similar with those of NA group. In PA treated groups, only small part of smear layer remained on the post spaces. Coronal region showed cleaner dentin surfaces than apical region with most of the dentin tubules exposed with wider opening of the dentinal tubule orifices (Fig. 5A and a). Compared with PA + BC group, PA + NA group showed higher level of demineralization on dentin surfaces with wider opening of the dentinal tubule orifices (Fig. 5). In the coronal region, intertubular dentins were demineralized apparently, showing large amount of characteristically widely opened dentinal tubules (Fig. 5B and b). The morphological presentations of PA + NEG group (Fig. 5C and c) and PA + NE group (Fig. 5D and d) were similar with those of PA + NA group.

## Discussion

In light of the results of this study, EGCG increased the bond strength and bond stability of fiber-post to NaOCl treated intraradicular dentin; SB2 showed higher bond strength than CSB regardless of root region and time. Thus, the null hypothesis was rejected.

Root canal is a narrow and sealed channel that can be hardly accessed by both endodontic instrument and various treating agents, which makes luting a fiber post to intraradicular dentin a problematic issue. NaOCl can dissolve the organics in smear layers, which is helpful for the removal of smear layers and can expose the dentinal tubules, thus producing a clean dentin surface and facilitating the bonding of resinous adhesives^20, 34^. According to the results of the present study, the effect of NaOCl pretreatment on post spaces on the bond strengths of fiber posts in root canal depended on the type of adhesives.

As shown in Fig. 4, a sole NaOCl irrigation is insufficient to remove the smear layer and expose the dentin tubules due to the absence in demineralization, which therefore contribute little to produce a dentin substrate suitable for well bonding. The side effect of remained NaOCl and its products on the polymerization of resinous adhesive may be the other important reason that contributes to the decrease in push-out strength of CSB. For CSB, the NaOCl treatment significantly decreased the push-out strengths of fiber posts in both coronal and apical root regions. This result is in accordance with a previous study^35^. NaOCl can break down to sodium chloride and oxygen. The oxygen and active NaOCl can lead to the formation of chloramine-derived radicals through oxidizing action^36, 37^, which would compete with the radicals generated from the resin adhesive and influence the polymerization of adhesive^38^. Micro-Raman spectroscopy could assess the adhesive degrees of conversion (DC) based on the intensity variation of the peak at 1640 cm^−1^ (C=C in methacrylate) relative to the peak corresponding to the C=C bond in the aromatic ring (1608 cm^−1^). The results of DC evaluation in the present study confirmed the negative effect of NaOCl on the polymerization of CSB. Post space pretreatments with NaOCl significantly decreased the DC of CSB. The side effect of NaOCl on the polymerization of resin has already been reported in literature^23, 26^. As far as we know, previous published studies used attenuated total reflectance-fourier transform infrared spectroscopy (ATR-FTIR) to evaluate the DC of adhesives^39^, an﻿d﻿ the present study is the first to use a real dentin-bonding model to evaluate the effect of NaOCl on the polymerization of adhesive resin.

For SB2, the NaOCl treatment slightly increased the push-out strength of fiber posts in root canal. SB2 is an etch-and-rinse adhesive. Previous studies have reported that pre-etching treatment was effective in the removal of the smear layer of debris or the sealer/gutta-percha remnants on the post space and improved the apical push-out strength of the fiber post^40^. Furthermore, the phosphoric acid etching after NaOCl treatment may remove the superficial dentin substrates that included the remained NaOCl and its products due to the demineralizing effect of phosphoric acid. That may also explain why the NaOCl treatment did not significantly decreased the DC and immediate bond strength of SB2 on the post space dentin as it did with CSB. Different from the results of the present study, da Cunha *et al*. found that NaOCl treatment decreased the bond strength of SB2 significantly^41^. In their study, the application of NaOCl was conducted after phosphoric acid etching, which is opposite to the procedure used in the clinical practice and manufactures’ instruction. This may be the reason why NaOCl treatment significantly decreased the bond strength of SB2 with intraradicular dentin in their study.

Chlorhexidine Gluconate (CHG) is an alternative irrigation solution to NaOCl because of its better range of antimicrobial activity and antimicrobial substantively^42^. CHG showed less toxicity and better antimicrobial activity than NaOCl at a low concentration^43^. Besides, former study proved that final irrigation with CHG reduced vertical root fracture resistance less than NaOCl did^44^. CHG also have been proved to inhibit the activity of dentin MMPs and improved the integrity of the hybrid layer in a 6-month clinical trial^45^. However, CHG could not neutralize the side-effect of NaOCl and some studies even have reported the occurrence of color change and precipitation when NaOCl and CHG are combined and might interfere with the seal of the root filling^46, 47^.

EGCG is a flavonoid produced in large amounts as a secondary metabolite by the Camellia sinensis plant, “green tea”^48^. It has been widely studied in Biology and Medical Science due to its excellent properties in the inhibition of viral or bacterial, incidences of cardiovascular diseases and cancer, decreasing fat absorption, and anti-aging and anti-inflammation^49, 50^. In the present study, using EGCG as final irrigants significantly increased the push-out strength and bond stability of fiber posts to NaOCl treated root for both CSB and SB2. For CSB, the immediate push-out strengths of NaOCl + EGCG groups were significantly higher than of Control groups. This should be attributed to the anti-oxidation ability of EGCG. EGCG treatment after the NaOCl irrigation could neutralize the residual NaOCl and oxygen through redox reaction and therefore restore the compromised bond strength of dentin after deproteinization with 5.25% NaOCl. The results of DC assessment showed that the DC of CSB in NEG groups were significantly higher than that in NA groups, confirming that EGCG treatment can reverse the negative effect of NaOCl on the polymerization of CSB. For SB2, NEG groups showed significantly highest push-out strengths than those of BC groups and NA groups. As we have discussed above, the combination usage of NaOCl and phosphoric acid may help to produce a clean dentin surface with exposed dentin tubules that suitable for bonding. However, the deproteinizing effect of NaOCl and demineralizing effect of phosphoric acid also produced over-demineralization of intertubular dentin and resulted in widely opened dentin tubules, as shown in Fig. 5. This kind of dentin substrate may produce a hybrid layer with resin monomers incompletely infiltrated into the wide dentin tubules and thus impair the bond strength^51^. The EGCG treatment after NaOCl irrigation is capable of neutralizing the remained NaOCl and stopped the damaging erosion effect of NaOCl. That may be the one reason for the high push-out strengths in NEG groups of SB2. A former study conducted by Du *et al*. reported that additional use of EGCG could improve the dentin bond strength and bond durability of SB2^52^. The authors explained that EGCG was a strong anti-oxidant and allowed free-radical polymerization of the adhesive to proceed without premature termination.

MMP inhibitors have been suggested as a useful tool in the prevention of the degradation of incompletely resin-infiltrated collagen fibrils caused by MMPs in the HL, thus increasing the longevity of dentin-adhesive interfaces^53^. Du *et al*. suggested that the MMPs inhibiting effect of EGCG should be responsible for the promising effect of EGCG on the bond stability. Different from *Du*’*s* study^52^, EGCG was dissolved in the anhydrous ethanol first and used as a separate treatment solution before the application of the adhesive in the present study. Firstly, the direct addition of EGCG into the adhesive might have some side effects on the physical and chemical properties of the adhesive and thus impair the bonding properties^54^. Besides, in the present study, EGCG can contact with the fiber collagen and the exposed MMPs directly, thus promoting the inhibiting effect of EGCG on MMPs. However, further study should be done to find the exact mechanism of EGCG on keeping the bond stability.

Previous studies reported that the ethanol pretreatment on the dentin surface (called as an ethanol-wet bonding technique) improved the dentin bond strength and bond stability of hydrophilic adhesive SB2^55^. Researchers believed that ethanol could take the place of remained water and support the demineralized dentin collagen matrix. The hydrophilic adhesive had better miscibility with ethanol-saturated collagen matrix, leading to better adhesive resin infiltration and strong bond strength^55^. Present study used ethanol as solvent to prepare EGCG solution. For CSB, NE groups failed to increase the bond strength and had significantly lower bond stability than NEG groups. As ethanol could not neutralize the residual NaOCl, the insufficiently polymerized adhesive would lead to low bond strength and poor bond stability. For SB2, NEG groups had highest bond strength and best bond stability than other groups. Though etch and rinse procedure could partly eliminate the side effect of residual NaOCl, it still more or less compromises the structure of root dentin and polymerization of adhesive. The final irrigation of EGCG seemed to be essential. A recent published study combined EGCG and ethanol-wet bonding together as a new pretreating technique before bonding, proved effective on improving immediate dentin bond strength and bond stability^56^. Therefore, using EGCG as a final irrigants showed double effects on neutralizing the side effects of NaOCl and increasing the bond stability.

## Conclusion

The effect of post space pretreatment with NaOCl on the bonding to intraradicular dentin depended on the type of adhesive. However, using EGCG as final irrigation increased the push-out strength and bond stability of fiber post to NaOCl treated intraradicular dentin for both self-etching adhesive CSB and etch-and-rinse adhesive SB2 regardless of root region.

## Materials and Methods

### Specimen Preparation

EGCG was dissolved in anhydrous ethanol at the concentration of 400 μg/mL to prepare the EGCG irrigants. Two hundred and twenty-four human premolars with single and straight root canal of similar length were used in this study. All teeth were collected after the patients’ informed consent and the protocol employed was approved by the Ethic Committee for Human Studies of the Fourth Military Medical University. All the experiments were carried out in accordance with the approved guidelines and regulations.

The teeth were stored in 1% chloramine T (chloramine T, Sigma–Aldrich Co., St. Louis, USA) at 4 °C within one month before using. Soft tissues and calculus were removed by scalers (Cattoni Scaler, Hu Friedy. Mfg Co., LLC, Chicago, USA) and cleaned with ultrasonic (UC50D, BioSonic, Coltene Whaledent, Inc., Ohio, USA). All teeth were decoronated at the cemento–enamel junction using a low-speed diamond saw (SYJ-150; MTI Corp., Shenyang, China) to get roots with exposed root canals. The canals of the teeth were instrumented to a working length, 1 mm from the apex, using an ISO 35 size master apical file (Dentsply Maillefer, Ballagues, Switzerland). A step-back technique was performed to prepare the canals using K-type files (Dentsply Maillefer, Ballagues, Switzerland) and Gates Glidden drills (ISO size 70–90; Dentsply Maillefer). Between the use of each file, canals were irrigated with 1 mL of 5.25% NaOCl (Sodium Hypochlorite, Kermel Chemical Reagent Co., Inc., Tianjin, China) and 17% ethylenediamine tetracetic acid (EDTA, Guoan biotechnology Co., Ltd., Xi’an, China) was used as the final rinse. After completely dried with absorbent paper points (Absorbent Paper Points; Meta Biomed Co., LTD, Baotou, China), the canals were obturated with gutta-percha points (Meta Biomed Co., Seoul, Korea) and AH-Plus Sealer (Dentsply DeTrey GmbH, Konstanz, Germany) using a lateral condensation technique. After removing excessive coronal gutta-percha, the coronal orifices of root canals were sealed with sticky wax and stored in water at 37 °C.

After 72 hours (h) water storage, 9 mm of gutta-percha was removed using a universal drill (3 M ESPE, St. Paul, MN, USA), leaving an apical seal of more than 4 mm. The post space was prepared in each root using a Rely X Fiber Post drill of size 3 (3 M ESPE, St. Paul, MN, USA). All prepared roots were firstly randomly divided into four groups according to the four post space pretreatments using different irrigants:

(i) Black control group (referred as BC group): post spaces were irrigated with 10 mL distilled water for 1 minute (min); (ii) NaOCl irrigation group (referred as NA group): post spaces were irrigated with 10 mL 5.25% NaOCl for 1 min, then stop with 10 mL distilled water for 1 min; (iii) NaOCl + EGCG group (referred as NEG group): post spaces were irrigated with 10 ml 5.25% NaOCl for 1 min, followed with 10 mL distilled water, and 10 ml EGCG irrigant for 1 min as final irrigation; (iv) NaOCl + Ethanol group (referred as NE group): post spaces were irrigated with 10 ml 5.25% NaOCl for 1 min, followed with 10 mL distilled water, and 10 ml anhydrous ethanol solution for 1 min as final irrigation.

All irrigants were introduced into root canals using a 10-ml plastic syringe with a 25-gauge tip (Sterile Hypodermic Syringes, Weigao Group Medical Polymer CO., LTD, Shandong, China). The excessive irrigants and water were removed with paper points.

### Push-out test

Thirty-two treated roots in each group were then divided into two subgroups according to the two bonding systems used (sixteen for each): (1) two-step self-etching adhesive Clearfil SE Bond (Kuraray Medical, Tokyo, Japan), referred as CSB; and (2) two-step etch-and-rinse adhesive Single Bond 2 (3 M ESPE, St. Paul, MN, USA), referred as SB2. The roots in each subgroup were treated with the corresponding adhesive strictly according to the manufactures’ instruction (Table 4).

**Table 4:** Adhesives and cement used in this study.

Materials	Composition	Application procedures
Adper Single Bond 2 (3 M ESPE, St Paul MN)	Bis-GMA; HEMA; Polyalkenoic; Acid copolymer; Photoinitiators; Ethanol; Water.	Phosphoric acid-etching for 15 s; Rinse with water for 10 s; Dry with paper points; 2 coats of adhesive were applied; Air-dried for 5 s; Light cure for 10 s.
Clearfil SE Bond (Kuraray Medical, Okayama, Japan)	SE-Primer: 10-MDP; HEMA; Hydrophilic dimethacrylate; Dl-Camphorquinone; N,N-diethanol-p-toluidine; Water.	Apply primer for 20 s; Gentle air blow; Apply bond for 5 s; Gentle air blow; Light cure for 10 s.
	SE-Bond:10-MDP; Bis-GMA; HEMA; Hydrophobic dimethacrylate; Dl-Camphorquinone; N,N-diethanol-p-toluidine; Silanated Colloidal silica.	
Clearfil DC Core (Kuraray Medical, Okayama, Japan)	10- MDP; Bis-GMA; HEMA; Hydrophilic aliphatic dimethacrylate; Hydrophobic aliphatic methacrylate; Colloidal silica; Sodium fluoride; Dl-Camphorquinone; Accelerators; Initiators; Ethanol; Water	Squeeze paste from the syringe directly into the root canal; Insert the post into the canal within 1 min after application of paste; Cure paste using the dental curing unit.

Bis-GMA: bisphenol-A diglycidyl ether dimethacrylate; HEMA: 2-hydroxyethyl methacrylate; MDP: 10-methacryloyloxydecyl dihydrogen phosphate.

After adhesive treatments, the roots from each subgroup were cemented with a fiber post (3 M ESPE, St. Paul, MN, USA) using a dual-cure composite resin cement (Clearfil DC Core Auto-mix; Kuraray Medical, Tokyo, Japan). The cement was introduced into the adhesive treated post space with an auto-mix cartridge and syringe provided by the manufacture. The fiber post was taken as manufactured. The posts was coated with the well-mixed cement and slowly inserted into full depth of the post space with finger pressure. The cement was then light-polymerized by a light emitting diode (LED) curing light (Elipar S10, 3 M ESPE, St. Paul, MN, USA) for 40 seconds (s) from four directions of the occlusal surface (buccal, palatal, mesial, and distal) respectively to make sure the cement be completely cured. The power density of the light was maintained at 1200 mW/cm^2^. Eight bonded roots from each subgroup were subjected to push-out test using the method has been previously reported^26^. All specimens were stored at 37 °C and 100% relative humidity for 24 h^13^. Eight roots from each group were used to conduct push-out bond strength test after thermocycling for 10,000 cycles and the other eight were used to conduct immediate push-out bond strength test. The roots were sectioned perpendicularly to the long axis of post under water-cooling with low speed saw to obtain seven slices of 1 mm thickness. The first slice from the top was excluded. Thus, 6 slices were included for each root canal. The coronal three slices were considered to represent the coronal root region, and the apical three slices were considered to represent the apical root region. Totally 48 slices for each group were available for push-out test, consisting of 24 slices from the coronal or apical region dividedly. The sample size of push-out strength was determined by preliminary power analysis, which revealed that having at least 19 specimens per final experimental group would assure the power of 90% for finding the statistical significance of four factors as well as the significance of their interactions in a four-way (2 × 2 × 2 × 4) ANOVA design, at a 0.001value of type 1 errors. The difference of 3 MPa for push-out strength was assumed as relevant according to our pilot study. The calculations were handled by PASS Power Analysis Software, version 11.0 (NCSS; Kaysville, UT, USA).

The slice was then fixed on the metal support of a universal testing machine (AGS-10-KN; Shimadzu, Kyoto, Japan) with the apical aspect facing the loading plunger. The plunger was fixed so that it only contacted the post during loading and thus distributing the shear stresses along the bonded interface. The load was applied from the apical end of the root slice to coronal end of the slice and in an apical–coronal direction, thus avoiding any limitation to post movement. The bonding strength was calculated by dividing the load in Newton (N) by the area of the bonded interface. The area was calculated using the equation: S = π (R + r) [(h^2+^ (R – r)^2^]^0.5^, where R is the coronal post radius, r is the apical post radius, h is the thickness of root slice^13^. The R, r and h values of each slice were individually measured with a digital caliper (605-04, Harbin Measuring & Cutting Tool Group Co., Ltd, Harbin, China) with 0.01 mm accuracy.

The fracture surfaces were evaluated by an optical microscope (SMZ1500; Nikon, Japan) at 110 magnification and classified into five groups using the criteria according to previous studies^9^: (i) adhesive failure between post and cement; (ii) adhesive failure between dentin and cement; (iii) cohesive failure within cement; (iv) cohesive failure within dentin; and (v) mixed failure.

### Micro-Raman analysis for degrees of conversion (DC) measurement

Twenty roots from each group with different post space pretreatment were used for degrees of conversion (DC) measurement. In each group, ten roots were split along the tooth axis in the linguo-buccal direction using a chisel and a hammer to expose the entire extent of the root canal. The exposed post spaces were then divided into two subgroups and treated with two adhesives following the instruction in Table 3 but without light curing. The specimens were evaluated using a micro-Raman spectrometer (HR800, Horiba JOBIN YVON, Paris, France) with a laser wave length of 633 nm and exposure time of 60 s to acquire the reference (r_1608cm_^−1^) and reaction (r_1640cm_^−1^) peaks of CSB and SB2 before curing. The spectra were calibrated with known lines of silicon before each measurement. The laser was focused through a 50-magnification objective of the matched microscope. Five random positions of each group were tested. Data were acquired over the spectral region from 100 to 2000 cm^−1^ and analyzed with software Origin software, version 9.0 (OriginLab Corporation, Northampto, USA). The average values of five positions were taken as the final values.

The other ten roots were also divided into two subgroups and luted with a fiber post using the two adhesives as described in section of “Push-out test”. After been stored at 37 °C and 100% relative humidity for 24 h^41^, each specimen was then cut into two parts along the direction of the axis of the fiber post using a low speed saw under water cooling. Exposed bonding interfaces of each part was ground flat using silicon carbide paper up to 4000-grit and then polished with diamond paste down to 0.25 µm. The polished surfaces were analyzed by acquiring spectra in five line-scans at random position, starting from the resin cement and ending in the sound mineralized dentin. One Raman spectrum was collected every 2 μm vertical to the dentin-adhesive interface using a computer-controlled motorized x-y-z stage, with an exposure time of 60 s and laser wave length of 633 nm. The reference (R_1608cm_^−1^) and reaction (R_1640cm_^−1^) peaks of cured CSB and SB2 were acquired from the five line scans and the average values were taken as the final values.

The ratio between the two peaks was then acquired as previous studies^41^ and the DC was calculated following the equation^41^: DC (%) = [1 − (R_1640cm_^−1^ **/**R_1608cm_^−1^)**/**(r_1640cm_^−1^ **/**r_1608cm_^−1^)] × 100 (%), where R_1640cm_^−1^ and R_1608cm_^−1^ are the Raman intensity of aliphatic and aromatic band after the adhesives been cured, r_1640cm_^−1^ and _1608cm_^−1^ are the Raman intensity of aliphatic and aromatic band before the adhesives been cured.

### SEM observation

Four roots from each group after different post space pretreatments were used for SEM assessment to observe the morphologic patterns of the root canal surfaces. Two roots were observed without considering the effect of etching. The other two roots in each group were etched with 35% phosphoric acid gel (PA, Ultra-etch; Ultradent Products, South Jordan, UT, USA) for 15 s and rinsed with distilled water and dried with paper points, considering the pre-etching step when the etch-and-rinse adhesive SB2 being used. The PA treated roots from different groups were referred as PA + BC group, PA + NA, PA + NEG group and PA + NE group respectively. All roots were then split along the axis in the lingual-buccal direction using a chisel and a hammer to expose the entire extent of the post space. The exposed post spaces were then gold-sputtered (Hitachi E-1045, Tokyo, Japan) and observed under SEM (SEM, Hitachi FE-SEM 4800, Tokyo, Japan). The SEM microphotographs were taken at 2 mm and 6 mm levels from the cemento–enamel junction of each root, and identified as coronal and apical portion of post spaces respectively.

### Statistical analysis

The data of push-out test and DC evaluation were first verified by the Kolmogorov–Smirnov test for their normal distribution and by Levenes test for the homogeneity of variances. The results of push-out strengths were analyzed with four-way ANOVA with push-out strength as the dependent variable and post space pretreatment, type of adhesive, root region, and time as fixed factors. The results of DC were analyzed using two-way ANOVA. Tukey test was used for post hoc comparisons. The data of failure mode after push-out test were analyzed using the chi-square test. In all the tests, the level of significance was analyzed with SPSS software, version 17.0 (SPSS, Chicago, IL, USA).

## References

[1] Schwartz RS, Robbins JW (2004). Post placement and restoration of endodontically treated teeth: a literature review. J Endodon..

[2] Baba NZ, Golden G, Goodacre CJ (2009). Nonmetallic prefabricated dowels: a review of compositions, properties, laboratory, and clinical test results. J Prosthodont..

[3] Cagidiaco MC, Goracci C, Garcia-Godoy F, Ferrari M (2008). Clinical studies of fiber posts: a literature review. Int J Prosthodont..

[4] Dietschi D, Duc O, Krejci I, Sadan A (2008). Biomechanical considerations for the restoration of endodontically treated teeth: a systematic review of the literature, Part II (Evaluation of fatigue behavior, interfaces, and *in vivo* studies). Quintessence Int..

[5] Mastoras K, Vasiliadis L, Koulaouzidou E, Gogos C (2012). Evaluation of push-out bond strength of two endodontic post systems. J Endod..

[6] Cantoro A (2011). Retentive strength and sealing ability of new self-adhesive resin cements in fiber post luting. Dent Mater..

[7] Goracci C, Ferrari M (2011). Current perspectives on post systems: a literature review. Aust Dent J..

[8] Ferrari M, Mannocci F, Vichi A, Cagidiaco MC, Mjor IA (2000). Bonding to root canal: structural characteristics of the substrate. Am J Dent..

[9] Serafino C, Gallina G, Cumbo E, Ferrari M (2004). Surface debris of canal walls after post space preparation in endodontically treated teeth: a scanning electron microscopic study. Oral Surg Oral Med Oral Pathol Oral Radiol Endod.

[10] Ferrari M, Vichi A, Grandini S, Goracci C (2001). Efficacy of a self-curing adhesive-resin cement system on luting glass-fiber posts into root canals: an SEM investigation. Int J Prosthodont..

[11] Shafiei F, Alikhani A, Alavi AA (2013). Effect of chlorhexidine on bonding durability of two self-etching adhesives with and without antibacterial agent to dentin. Dent Res J..

[12] Li XJ (2014). Effect of luting cement and thermomechanical loading on retention of glass fibre posts in root canals. J Dent..

[13] Santos J (2009). Determination of matrix metalloproteinases in human radicular dentin. J Endod..

[14] Tjaderhane L (2013). Optimizing dentin bond durability: control of collagen degradation by matrix metalloproteinases and cysteine cathepsins. Dent Mater..

[15] Stanislawczuk R (2014). Effects of chlorhexidine-containing adhesives on the durability of resin-dentine interfaces. J Dent..

[16] Coniglio I (2008). Post space cleaning using a new nickel titanium endodontic drill combined with different cleaning regimens. J Endod..

[17] Breschi L (2008). Dental adhesion review: aging and stability of the bonded interface. Dent Mater..

[18] Goracci C, Sadek FT, Fabianelli A, Tay FR, Ferrari M (2005). Evaluation of the adhesion of fiber posts to intraradicular dentin. Oper Dent..

[19] Haapasalo M, Shen Y, Qian W, Gao Y (2010). Irrigation in endodontics. Dent Clin North Am..

[20] Cecchin D, Almeida JF, Gomes BP, Zaia AA, Ferraz CC (2012). Deproteinization technique stabilizes the adhesion of the fiberglass post relined with resin composite to root canal. J Biomed Mater Res B Appl Biomater..

[21] Barbosa de Souza F, Silva CH, Guenka Palma Dibb R, Sincler Delfino C, Carneiro de Souza Beatrice L (2005). Bonding performance of different adhesive systems to deproteinized dentin: microtensile bond strength and scanning electron microscopy. J Biomed Mater Res B Appl Biomater..

[22] Abo T, Asmussen E, Uno S, Tagami J (2006). Short- and long-term in vitro study of the bonding of eight commercial adhesives to normal and deproteinized dentin. Acta Odontol Scand..

[23] Nikaido T, Takano Y, Sasafuchi Y, Burrow MF, Tagami J (1999). Bond strengths to endodontically-treated teeth. Am J Dent..

[24] Acquaviva PA (2009). Degree of conversion of three composite materials employed in the adhesive cementation of indirect restorations: a micro-Raman analysis. J Dent..

[25] Weston CH, Ito S, Wadgaonkar B, Pashley DH (2007). Effects of time and concentration of sodium ascorbate on reversal of NaOCl-induced reduction in bond strengths. J Endod..

[26] Vongphan N, Senawongse P, Somsiri W, Harnirattisai C (2005). Effects of sodium ascorbate on microtensile bond strength of total-etching adhesive system to NaOCl treated dentine. J Dent..

[27] Lai SC (2002). Reversal of compromised bonding in bleached enamel. J Dent Res..

[28] Lai SC (2001). Reversal of compromised bonding to oxidized etched dentin. J Dent Res..

[29] Puri, A., Nguyen, H. X. & Banga, A. K. Microneedle-mediated intradermal delivery of epigallocatechin-3-gallate. *Int J Cosmet Sci* (2016). 10.1111/ics.12320 27009797

[30] Chowdhury A, Sarkar J, Chakraborti T, Chakraborti S (2015). Role of Spm-Cer-S1P signalling pathway in MMP-2 mediated U46619-induced proliferation of pulmonary artery smooth muscle cells: protective role of epigallocatechin-3-gallate. Cell Biochem Funct..

[31] Zhang SC, Kern M (2009). The role of host-derived dentinal matrix metalloproteinases in reducing dentin bonding of resin adhesives. Int J Oral Sci..

[32] Sartor L (2002). Inhibition of matrix-proteases by polyphenols: chemical insights for anti-inflammatory and anti-invasion drug design. Biochem Pharmacol..

[33] Demeule M, Brossard M, Page M, Gingras D, Beliveau R (2000). Matrix metalloproteinase inhibition by green tea catechins. Biochim Biophys Acta..

[34] Perdigao J, Thompson JY, Toledano M, Osorio R (1999). An ultra-morphological characterization of collagen-depleted etched dentin. Am J Dent..

[35] Elnaghy AM (2014). Effect of QMix irrigant on bond strength of glass fibre posts to root dentine. Int Endod J..

[36] Daumer KM, Khan AU, Steinbeck MJ (2000). Chlorination of pyridinium compounds. Possible role of hypochlorite, N-chloramines, and chlorine in the oxidation of pyridinoline cross-links of articular cartilage collagen type II during acute inflammation. J Biol Chem..

[37] Hawkins CL, Davies MJ (1999). Hypochlorite-induced oxidation of proteins in plasma: formation of chloramines and nitrogen-centred radicals and their role in protein fragmentation. Biochem J..

[38] Monticelli F, Toledano M, Silva AS, Osorio E, Osorio R (2008). Sealing effectiveness of etch-and-rinse vs self-etching adhesives after water aging: influence of acid etching and NaOCl dentin pretreatment. J Adhes Dent..

[39] Ruyter IE, Oysaed H (1982). Conversion in different depths of ultraviolet and visible light activated composite materials. Acta Odontol Scand..

[40] Zhang L (2008). Effect of post-space treatment on retention of fiber posts in different root regions using two self-etching systems. Eur J Oral Sci..

[41] da Cunha LF, Furuse AY, Mondelli RF, Mondelli J (2010). Compromised bond strength after root dentin deproteinization reversed with ascorbic acid. J Endod..

[42] Oncag O (2003). Comparison of antibacterial and toxic effects of various root canal irrigants. Int Endod J..

[43] Zargar N, Dianat O, Asnaashari M, Ganjali M, Zadsirjan S (2014). The effect of smear layer on antimicrobial efficacy of three root canal irrigants. Iranian Endodontic Journal..

[44] Sungur, D. D., Altundasar, E., Uzunoglu, E. & Yilmaz, Z. Influence of different final irrigation regimens and various endodontic filling materials on vertical root fracture resistance. *Nigerian Journal of Clinical Practice*. **19** (2015). 10.4103/1119-3077.164334 26856293

[45] Hebling J, Pashley DH, Tjäderhane L, Tay FR (2005). Chlorhexidine arrests subclinical degradation of dentin hybrid layers *in vivo*. Journal of Dental Research..

[46] Basrani BR, Manek S, Sodhi RN, Fillery E, Manzur A (2007). Interaction between sodium hypochlorite and chlorhexidine gluconate. Journal of Endodontics..

[47] Zehnder M (2006). Root Canal Irrigants[J]. Journal of Endodontics..

[48] Pharn-Huy LAN, He H, Phamhuy C (2008). Green tea and health: An overview. J Food Agric Environ.

[49] Steinmann J, Buer J, Pietschmann T, Steinmann E (2013). Anti-infective properties of epigallocatechin-3-gallate (EGCG), a component of green tea. Br J Pharmacol..

[50] Huo C (2008). The challenge of developing green tea polyphenols as therapeutic agents. Inflammopharmacology.

[51] Hashimoto M (2000). The effect of hybrid layer thickness on bond strength: demineralized dentin zone of the hybrid layer. Dent Mater..

[52] Du X, Huang X, Huang C, Wang Y, Zhang Y (2012). Epigallocatechin-3-gallate (EGCG) enhances the therapeutic activity of a dental adhesive. J Dent..

[53] Liu RR (2014). Anti-proteolytic capacity and bonding durability of proanthocyanidin-biomodified demineralized dentin matrix. Int J Oral Sci..

[54] Xiao YH (2009). Antibacterial activity and bonding ability of an adhesive incorporating an antibacterial monomer DMAE-CB. J Biomed Mater Res B Appl Biomater..

[55] Carvalho CA (2009). Effect of ethanol application on post-luting to intraradicular dentine. Int Endod J..

[56] Yang H, Guo J, Deng D, Chen Z, Huang C (2016). Effect of adjunctive application of epigallocatechin-3-gallate and ethanol-wet bonding on adhesive-dentin bonds. J Dent..

